# Low-dose quercetin positively regulates mouse healthspan

**DOI:** 10.1007/s13238-019-0646-8

**Published:** 2019-07-19

**Authors:** Lingling Geng, Zunpeng Liu, Si Wang, Shuhui Sun, Shuai Ma, Xiaoqian Liu, Piu Chan, Liang Sun, Moshi Song, Weiqi Zhang, Guang-Hui Liu, Jing Qu

**Affiliations:** 1 Advanced Innovation Center for Human Brain Protection, National Clinical Research Center for Geriatric Disorders, Xuanwu Hospital Capital Medical University, Beijing, 100053 China; 2 State Key Laboratory of Membrane Biology, Institute of Zoology, Chinese Academy of Sciences, Beijing, 100101 China; 3 State Key Laboratory of Stem Cell and Reproductive Biology, Institute of Zoology, Chinese Academy of Sciences, Beijing, 100101 China; 4 Key Laboratory of Genomics and Precision Medicine, Beijing Institute of Genomics, Chinese Academy of Sciences, Beijing, 100101 China; 5 University of Chinese Academy of Sciences, Beijing, 100049 China; 6 National Laboratory of Biomacromolecules, CAS Center for Excellence in Biomacromolecules, Institute of Biophysics, Chinese Academy of Sciences, Beijing, 100101 China; 7 The MOH Key Laboratory of Geriatrics, Beijing Hospital, National Center of Gerontology, Beijing, 100730 China; 8 Institute for Stem Cell and Regeneration, Chinese Academy of Sciences, Beijing, 100101 China; 9 Beijing Institute for Brain Disorders, Beijing, 100069 China

**Dear Editor,**

Aging is the leading risk factor for many chronic diseases, accounting for almost 60% of all deaths worldwide. How to achieve healthy aging, alleviate aging-related diseases, and extend healthspan has become a main topic of biomedical research (He et al., 2019). Geroprotective compounds, such as metformin and rapamycin, have been shown to improve both healthspan and lifespan in mice (Martin-Montalvo et al., 2013; Bitto et al., 2016), whereas nicotinamide partially improves healthspan in mice (Mitchell et al., 2018). In addition, senolytics, compounds that eliminate senescent cells, have been proven to improve physical function and increase lifespan in mice (Xu et al., 2018). Although none have proven to be clinically reliable in delaying aging or treating frailty in humans, these compounds have already provoked enthusiasm for identifying a potential “elixir”. Therefore, the exploration of more geroprotective compounds, especially natural active compounds, holds great potential for the development of geriatric medicines.

Quercetin (Que) is a natural bioflavonoid found in fruits and vegetables such as apples and onions. Que (50 mg/kg) in combination with dasatinib (5 mg/kg) (abbreviated as D + Q) has been shown to effectively eliminate senescent cells via induction of apoptosis, thus alleviating senescence-related phenotypes and improving physical function and lifespan in mice (Zhu et al., 2015; Xu et al., 2018). In addition, Que (10 mg/kg) in combination with dasatinib (5 mg/kg) has been reported to reduce hepatic steatosis (Ogrodnik et al., 2017). In each of these *in vivo* studies, however, Que was used at high doses ranging from 10 to 50 mg/kg body weight, which raises concerns about dose-dependent side effects such as headaches and limb tingling (Shoskes et al., 1999). As a selective tyrosine kinase receptor inhibitor, dasatinib is associated with warnings and precautions including pulmonary arterial hypertension and low blood cell counts. Therefore, high-dose Que and extra side effects of dasatinib would hamper potential clinical applications of Que in geriatric medicines. Through natural products screening using Werner syndrome (WS) human mesenchymal stem cells (hMSCs), we recently identified Que as a geroprotective agent that counteracts accelerated and natural aging of hMSCs at a concentration of as low as 100 nmol/L, which is 100 times lower than the concentration of Que (10 μmol/L) previously used in combination with dasatinib as senolytic drugs to eliminate senescent cells in human umbilical vein cells (HUVECs) (Zhu et al., 2015; Geng et al., 2018).

To explore the geroprotective effect of low-dose Que monotherapy in rodents, we evaluated the *in vivo* effect of long-term low-dose Que administration under physiological-aging condition. Que was given to 14-month-old C57BL/6J male mice by weekly oral gavage at a concentration of 0.125 mg/kg body weight, which is 80–400 times lower than that of the previously tested D + Q (10–50 mg/kg body weight) regimens (Fig. 1A), with vehicle (10% PEG400 in PBS)-treated mice as controls (Zhu et al., 2015; Xu et al., 2018). After eight months of treatment, Que-treated mice showed decreased hair loss with normal food intake, body weight, blood glucose and bone mineral density (Figs. 1B and S1A–D). Compared to vehicle-treated mice, mice subjected to Que treatment showed markedly improved exercise endurance in the RotaRod and treadmill tests, but normal grip strength by grip strength meter assay (Figs. 1C, 1D, and S1E–G). Accordingly, the cardiac function of these mice was examined by Doppler tissue imaging. Although ejection fraction (EF) and fractional shortening (FS) were unaffected, a higher frequency of the mitral ratio of peak early to late diastolic filling velocity (E/A) within the normal range was observed in Que-treated mice than in the age-matched controls (Figs. 1E and S1H). However, the lifespan was not prolonged by low-dose Que treatment observed up to the age of 31 months (Fig. S1I). Taken together, these data indicate that long-term low-dose Que administration alone sufficiently improves multiple aspects of healthspan, but not lifespan, in mice.

**Figure 1:**
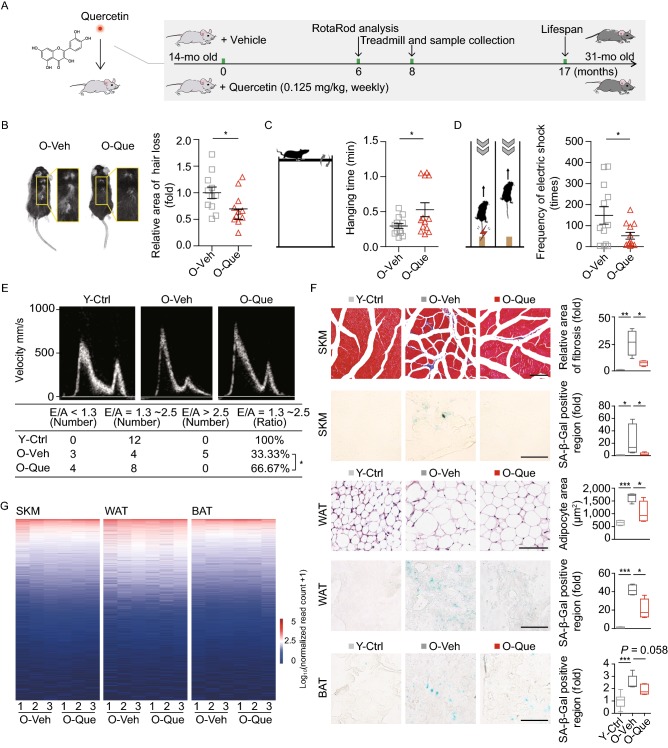
**Low-dose quercetin alone improved the healthspan of physiologically** **aging mice**. (A) Experimental design for drug administration. (B) Hair loss evaluation (*n* = 11). Data are shown as the mean ± SEM. *P < 0.05. (C) Hanging endurance on the RotaRod system (*n* = 13). Data are shown as the mean ± SEM. *P < 0.05. (D) Frequency of electric shock on the treadmill over 30 min (*n* = 12). Data are shown as the mean ± SEM. *P < 0.05. (E) The ratio of peak velocity of early to late filling of mitral inflow (E/A) (*n* = 12). The table shows the number of mice in 3 kinds of E/A ranges, and Que treatment increased the ratio of normal E/A mice. *P < 0.05. (F) Masson’s trichrome staining in SKM showed moderate perivascular and interstitial fibrosis (blue areas) (*n* = 4). Data are shown as the mean ± SEM. *P < 0.05. Scale bar, 100 μm. SA-β-Gal staining analysis of SKM, WAT and BAT. Scale bar, 100 μm (*n* = 4). Data are shown as the mean ± SEM. ***P < 0.001, **P < 0.01, *P < 0.05. Haematoxylin and eosin staining of WAT. Scale bar, 100 μm (*n* = 4). Data are shown as the mean ± SEM. *P < 0.05. (G) Global gene expression profiling in SKM, WAT and BAT (*n* = 3). Y-Ctrl represents 10-week-old young male mice, and O-Veh and O-Que represent vehicle (10% PEG400 in PBS)- or low-dose Que-treated old male mice

To investigate how Que improved healthspan in mice, we collected 11 different kinds of tissues from 10-week young male mice (Y-Ctrl) and vehicle (O-Veh)- and low-dose Que-treated 22-month old male mice (O-Que). No significant difference was observed in organ weights between O-Veh and O-Que (Fig. S2A). Given that exercise endurance and diastolic function were improved by Que, we particularly examined the changes in skeletal muscles (SKM), white adipose tissues (WAT), brown adipose tissues (BAT) and hearts. Upon Que treatment, the arrangement of muscle fibers became more regular and compact with less fibrosis and senescence (Figs. 1F and S2B). In WAT, the increases in adipocyte size and senescence-associated β-galactosidase (SA-β-Gal)-positive area during aging were both alleviated upon Que treatment (Fig. 1F). In BAT, although adipocyte size was unaffected, there was a decreasing trend of the SA-β-Gal-positive area upon Que treatment (Figs. 1F and S2B). By comparison, we did not observe any significant differences in mouse hearts by histological analysis and SA-β-Gal staining (Fig. S2B). Therefore, these data suggest that long-term low-dose Que administration may delay aging of SKM, WAT, and BAT in mice.

To further explore the molecular mechanisms of the beneficial effects of Que, we performed whole-transcriptome RNA sequencing (RNA-seq) of SKM, WAT, and BAT from Y-Ctrl, O-Veh, and O-Que mice. Global gene expression profiling revealed that most protein coding genes were unaffected after long-term low-dose Que administration (Fig. 1G). Accordingly, we inferred that low-dose Que might exert its senostatic effect by regulating the expression of non-protein-coding RNAs.

We previously observed that Que alleviates hMSC senescence in part through the restoration of heterochromatin architecture in prematurely and physiologically aging hMSCs (Geng et al., 2018). Constitutive heterochromatins are predominantly comprised of repetitive elements (REs), including retrotransposable elements (RTEs). The expression of RTEs is repressed via epigenetic regulation under normal conditions but is elevated during physiological aging, eliciting active transposition (De Cecco et al., 2013). Accordingly, mobilization of RTEs is likely to be a key contributor to tissue aging and cell degeneration (De Cecco et al., 2013). To investigate whether low-dose Que treatment antagonized the activation of RTEs, we examined the expression levels of various RTEs, including long terminal repeats such as LTR10C, LTR2C, LTR35A, and LTR3B, and non-long terminal repeats including long interspersed nuclear elements 1 (L1, also known as LINE-1) and short interspersed nuclear elements (SINEs) such as Alu in WS hMSCs after continuous Que treatment at the concentration of 100 nmol/L. Que treatment silenced the transcription of various RTEs in WS hMSCs, consistent with the rejuvenated cellular phenotypes (Fig. 2A). To test whether Que treatment may also repress activation of RTEs in a mouse *in vivo* model, we compared the transcriptional levels of RTEs such as L1, SINE B1, LTR41, LTR42 and MLV5 in multiple tissues of Y-Ctrl, O-Veh, and O-Que mice. Consistently, most RTEs were transcriptionally upregulated in the SKM and BAT of old mice compared to those of young mice and were repressed by Que treatment (Fig. 2B). Similar to the tendency in the BAT, RTEs in WAT from Que-treated mice were also slightly decreased (Fig. 2B). In line with enhanced L1 transcripts, there was an increased expression level of L1 open reading frame 1 protein (ORF1p) in SKM and BAT of aged mice, which could be reversed by long-term low-dose Que administration (Fig. 2C–E). These data indicate that Que represses RTE activation in senescent hMSCs and multiple aged mouse tissues.

**Figure 2:**
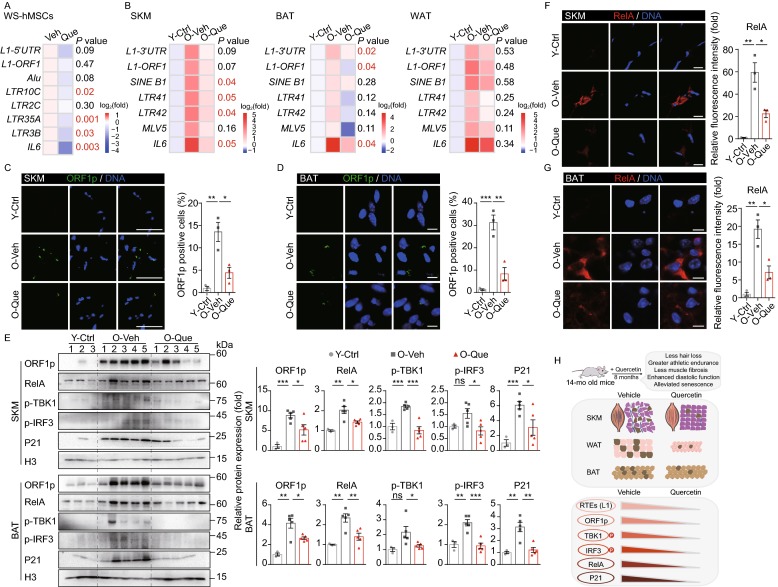
**Activation of retrotransposable elements (RTEs) was repressed in Que-treated WS hMSCs and certain mouse tissues**. (A) RT-qPCR analysis of RTEs in vehicle- and Que-treated WS hMSCs (passage 7) (*n* = 3). *P* values between vehicle and Que are shown on the right. (B) RT-qPCR analysis of RTEs in SKM, WAT and BAT of young male mice, old male mice treated with vehicle and Que (*n* = 4). *P* values between O-Veh and O-Que are shown on the right, *P* ≤ 0.05 were labeled in red. (C) Immunostaining of ORF1p in SKM of young male mice, old male mice treated with vehicle and Que (*n* = 3). Scale bar, 50 μm. Data are shown as the mean ± SEM (cell number ≥ 100). **P < 0.01, *P < 0.05. (D) Immunostaining of ORF1p in the BAT of young male mice, old male mice treated with vehicle and Que (*n* = 3). Scale bar, 7.5 μm. Data are shown as the mean ± SEM (cell number ≥ 100). ***P < 0.001, **P < 0.01. (E) Immunoblotting of ORF1p, RelA, p-TBK1, p-IRF3 and P21 in the SKM and BAT of young male mice (*n* = 3), old male mice treated with vehicle (*n* = 5) and Que (*n* = 5). ***P < 0.001, **P < 0.01, *P < 0.05, ns, not significant. (F) Immunostaining of RelA in the SKM of young male mice, old male mice treated with vehicle and Que (*n* = 3). Scale bar, 10 μm. Data are shown as the mean ± SEM (cell number ≥ 100). **P < 0.01, *P < 0.05. (G) Immunostaining of RelA in the BAT of young male mice, old male mice treated with vehicle and Que (*n* = 3). Scale bar, 7.5 μm. Data are shown as the mean ± SEM (cell number ≥ 100). **P < 0.01, *P < 0.05. Y-Ctrl represents 10-week-old young male mice, and O-Veh and O-Que represent vehicle (10% PEG400 in PBS)- or low-dose Que-treated old male mice. (H) A proposed model illustrating the senostatic effects of Que

In senescent cells, the activation of RTEs (such as L1) leads to genome instability and accumulation of cytosolic DNA that further binds to cytosolic sensor cGAS and activates TBK1 and IRF3, which subsequently promote senescence-associated secretory phenotype (SASP) (Takahashi et al., 2018; De Cecco et al., 2019). In addition, NF-κB/RelA in cGAS-STING-mediated NF-κB pathway acts with IRF3 and other transcription factors to induce the expression of inflammatory cytokines such as IL-6, the most prominent SASP cytokine (Chen et al., 2016). Notably, both p-TBK1 and p-IRF3 were increased in old mouse tissues compared to the young ones and were repressed upon Que treatment (Fig. 2E), indicating the effect of Que on inhibiting cGAS-STING pathway (Kato et al., 2017). Similarly, RelA (p65) was upregulated in aged mouse tissues and repressed upon Que treatment (Fig. 2E–G). Consistently, the inflammatory cytokine IL-6 was increased in old mice compared to young mice and Que antagonized the increase of IL-6 in both WS-hMSCs and old mouse SKM and BAT (Fig. 2A and 2B). Thus, our data suggest that Que may block SASP through the axis of heterochromatin-RTEs (L1)-innate immune response pathway (Fig. 2H).

In this study, we reported for the first time a geroprotective effect of low-dose quercetin alone that improved the healthspan of aged C57BL/6J male mice. Que-treated mice showed less hair loss, greater athletic endurance, enhanced diastolic function, and less muscle fibrosis, as well as alleviated cellular senescence in multiple tissues. Interestingly, these changes appear to be rarely associated with transcriptional alterations of protein-coding genes but are linked to heterochromatin stabilization and RTE silencing. Que treatment prevented L1 from hyperactivation, thereby inhibiting SASP. In contrast to the reported senolytic effect of high-dose D+Q (Xu et al., 2018), where Que exerts geroprotective effects via the induction of apoptosis of senescent cells, low-dose Que (0.125 mg/kg body weight) alone was sufficient to exert senostatic effects in mice by affecting heterochromatin stability through repression of RTEs activity in this study. In a translational context, low-dose Que monotherapy may be helpful to minimize the dose-dependent side effects compared to high-dose administration and avoid drug interference when used in combination, probably representing a potential therapeutic option for future clinical application (Shoskes et al., 1999). Of note, here we reported the geroprotective effect of Que in male mice and its effect remains to be studied in female mice.

The possible mechanism of low-dose Que in mice may be associated with its function as a heterochromatin stabilizer and its direct inhibitory activity against reverse transcriptase (Ono et al., 1990; Geng et al., 2018). Loss of heterochromatin architecture and genomic instability are two hallmarks of aging (Zhang et al., 2015). In advanced age, the expression of RTEs is often increased, which may in turn contribute to genomic instability and aging-associated cellular defects (De Cecco et al., 2013). Activation of L1 has been implicated in a variety of age-related disorders, including cancer and neurodegenerative diseases. The activation of L1 (and possibly other RTEs in mice) promotes the expression inflammatory factors, a feature of cellular senescence (De Cecco et al., 2019; Simon et al., 2019). Recently, it has been reported that nucleoside reverse-transcriptase inhibitors (NRTIs), such as lamivudine, stavudine, inhibit L1 retrotransposition and thus improve the healthspan and/or lifespan of SIRT6-knockout and physiologically aged mice (De Cecco et al., 2019; Simon et al., 2019). Que has been proven as a potential inhibitor of reverse transcriptase from Rauscher murine leukemia virus (RLV) and human immunodeficiency virus (HIV) by enzyme kinetic analysis, whereas its reverse transcriptase inhibition activity against RTEs in hMSCs and rodents has not been reported (Ono et al., 1990). Our data provide important evidence supporting the role of low-dose Que in safeguarding genomic stability (i.e. inhibition of retrotransposition), which at least in part contributes to its geroprotective activity in rodents.

## Electronic supplementary material

Below is the link to the electronic supplementary material.

Supplementary material 1 (PDF 438 kb)
