# Supplementary material for: Low-dose quercetin positively regulates mouse healthspan
Source: Protein Cell. 2019 Jul 19;10(10):770–5. doi: 10.1007/s13238-019-0646-8 (PMC6776572; doi:10.1007/s13238-019-0646-8)
Supplement: Supplementary file 1 — Supplementary material 1 (PDF 438 kb) [file 13238_2019_646_MOESM1_ESM.pdf]

MATERIALS AND METHODS

Mouse model and drug treatment

All animal experiments were performed according to protocols approved by the Chinese Academy of Science Institutional Animal Care and Use Committee. Wild-type C57BL/6J mice were maintained separately in a pathogen-free facility at 23–24 °C under a 12-hour light, 12-hour dark regimen with free access to normal chow and water. Cages and bedding were changed weekly. For Que treatment, quercetin (0.125 mg/kg) was administered via oral gavage with 100 µL of 10% PEG400 in PBS. For the lifespan study, 14-month-old male mice were treated with Que or vehicle (10% PEG400 in PBS) every week until death. Quercetin hydrate (Que, TCI, P0042) was used in this study. All other reagents were purchased from Sigma.

Cell culture

WS hMSCs were obtained as previously described (Geng et al., 2018; Zhang et al., 2015). The hMSCs were cultured in hMSC culture medium containing 90% α-MEM + Glutamax (Gibco), 10% fetal bovine serum (FBS, Gemcell, Lot A77E01F), 1 ng/mL FGF2 (Joint Protein Central) and 1% penicillin/streptomycin (Gibco). WS hMSCs were treated with vehicle (DMSO) or Que (100 nmol/L) from passage 5 and collected at passage 7 for RT-qPCR analysis (Geng et al., 2018). Quercetin hydrate (Que, TCI, P0042) was used.

Physical function measurements

Maximal hanging time was assessed using an accelerating RotaRod system (YLS-4C). Mice were acclimated on the RotaRod system once per day for 3 days. On the test day, mice were placed onto the RotaRod, which was started at 4 rpm. The rotating speed was accelerated from 4 to 40 rpm over a 5-minute interval. The time was recorded when the mice dropped off the RotaRod. The results were averaged from 3 trials. Forelimb grip strength (N) was determined using a grip strength meter (YLS-13A). The results were averaged over 10 trials. A Comprehensive Laboratory Animal Monitoring System was used to monitor food intake over a 24-h period (12-hour light and 12-hour dark). For treadmill performance, mice were trained on a treadmill at an incline of 5° (5-Lane Treadmills, LE8710MTS) over 3 days for 5 minutes each day, with the speed accelerated from 0 to 10 m/min. On the test day, the mice ran on the treadmill at an initial speed of 0 m/min, and then the speed was increased by 2 m/min to 10 m/min. The whole running time was set at 30 minutes. The frequency of mild electrical shock stimulus was recorded.

Doppler tissue imaging

Heart ejection fraction (EF, %) and fractional shortening (FS, %) and the ratio of peak velocity of early to late filling of mitral inflow (E/A) were tested by using a Doppler tissue imaging system (Vevo 2100). The mouse prothorax was first depilated, anesthetized with 2% isoflurane and placed on a 37 °C thermostat for ultrasound detection.

Mouse tissue sample collection and histological analysis

After 8 months of Que treatment, animals were euthanized, and 11 tissues were harvested and stored. Animals were perfused with normal saline, and

tissues were harvested and split into samples. SKM, WAT, BAT and hearts processed for RNA were flash frozen in cryotubes submerged in liquid nitrogen for further RNA-seq analysis. SKM and BAT processed for protein were flash frozen in cryotubes submerged in liquid nitrogen for further western blotting analysis. Samples processed for histology were fixed in 4% paraformaldehyde in PBS (4% PFA) overnight at 4 °C. Fixed tissues were washed with PBS and incubated with 30% sucrose in PBS overnight at 4 °C. Sucrose-treated tissues were embedded in paraffin or optimum cutting temperature compound (OCT). For haematoxylin and eosin staining, paraffin-embedded SKM, WAT and BAT were sectioned to a 7 μm thickness and stained with hematoxylin and eosin. Images were then captured and analyzed using ImageJ. For Masson's trichrome staining, paraffin-embedded SKM and hearts were sectioned to a 7 μm thickness, and sections were stained with Masson's trichrome stain according to the manufacturer's protocols. Images were captured and analyzed using ImageJ.

Senescence-associated β-galactosidase staining

Briefly, flash frozen OCT embedded tissue without 4% PFA fixation was cryosectioned at a 10 μm thickness, collected on superfrost plus microslides (VWR) and stored at −80 °C until use. Cryosections collected on slides were thawed and washed in PBS and fixed at room temperature for 5 minutes in 2% formaldehyde and 0.2% glutaraldehyde. Fixed tissues were then stained with freshly prepared staining solution for SA-β-Gal activity at 37 °C overnight. Images were taken, and the percentage of positive regions was calculated and analyzed using ImageJ.

RNA-seq data processing

SKM, WAT and BAT from Y-Ctrl, O-Veh and O-Que mice were snap-frozen in liquid nitrogen for RNA-seq analysis. The pipeline of RNA-seq data processing has been described previously (Geng et al., 2018). In brief, sequencing reads were trimmed and mapped to the UCSC mm10 mouse genome using hisat2 software (v2.0.4). The transcriptional expression level of each gene was counted by HTSeq (v0.6.1). Differentially expressed genes (DEGs) were computed using DESeq2 at the cutoff, adjusted P value (Benjamini-Hochberg) < 0.05 and absolute Log_2(fold change) ≥ 0.5. Heatmaps of the gene expression levels in four tissues were drawn based on the DESeq2 size factor normalized read count.

Quantitative real-time PCR (RT-qPCR)

Total RNA was extracted using TRIzol Reagent (Thermo Fisher Scientific). Total RNA (1-2 μg) was reverse transcribed to cDNA by using the GoScript Reverse Transcription System (Promega). Quantitative real-time PCR was performed with iTaq Universal SYBR Green Super mix (BioRad) on a CFX384 Real-Time PCR system (Bio-Rad). Data were normalized to β-actin transcript and calculated using the ΔΔCq method. Quantitative RT-PCR primers used are listed in Table S1 (De Cecco et al., 2013; De Cecco et al., 2019).

Immunofluorescence

Tissues of mice were embedded in optimal cutting temperature compound (OCT), flash-frozen, and stored at −80 °C. The OCT-embedded specimens

were cryosectioned at a 10 µm thickness using a Leica CM3050S cryomicrotome. The slides were fixed with 4% formaldehyde in PBS for 30 minutes and further permeabilized with 0.4% Triton X-100 in PBS for 1 hour at room temperature. Antibody incubation was preceded by a blocking step with 10% donkey serum in PBS for 1 hour at room temperature. Primary antibodies were diluted in the above blocking solution (1:100) and incubated overnight at 4 °C. The fluorescence-labeled secondary antibodies were also diluted in blocking solution and incubated for 1 hour at room temperature. Three 15-minute washing steps in PBS followed each antibody incubation. Hoechst 33342 (Thermo Fisher Scientific) was used to stain nuclear DNA.

Western blotting

Tissue samples were snap-frozen in cryotubes submerged in liquid nitrogen. When processed for western blotting analysis, the tissues were ground and lysed with 100 µL of RIPA buffer (0.1% SDS, 50 mmol/L Tris-HCl (pH 7.5), 1% NP-40, 0.5% sodium deoxycholate, 150 mmol/L NaCl) supplemented with $NaVO_4$, NaF and the protease-inhibitor mixture (Roche). After centrifugation at 13,000 rpm for 15 minutes, the supernatant was collected and stored at -80 °C. A BCA kit (Thermo Fisher Scientific) was used to measure the protein concentration. Twenty-five micrograms of proteins for each sample were separated by SDS-PAGE and transferred onto a PVDF membrane (Millipore). The membrane was incubated with primary antibodies and then HRP-conjugated secondary antibodies. The quantification was performed with Image Lab software for the ChemiDoc XRS system (Bio-Rad).

Antibodies and reagents

The primary antibodies for Western blotting were anti-LINE1-ORF1p (MABC1152, 1:1000) from Millipore, anti-P21 (2947, 1:1000) from Cell Signaling Technology, anti-p-TBK1 (Ser172, 5483, 1:1000) from Cell Signaling Technology, anti-p-IRF3 (Ser396, 29047S) from Cell Signaling Technology, anti-RelA (8242, 1:1000) from Cell Signaling Technology and anti-H3 (ab1791, 1:1000) from Abcam.

Primary antibodies for immunofluorescence staining were anti-LINE1-ORF1p (MABC1152, 1:100) from Millipore and anti-RelA (8242, 1:100) from Cell Signaling Technology.

Statistical analysis

Student's t-test was used for statistical analysis. Data are presented as the mean ± SEM. $P < 0.05$ was considered statistically significant.

Data availability

Raw sequencing data and processed data of RNA-seq have been deposited in the NCBI Gene Expression Omnibus (GEO) under the accession number GSE132129.

Figure S1. Lifespan and some physical functions were unaffected by Que. (A) Food intake (n = 11). Data are shown as the mean ± SEM. ns, not significant. (B) Body weight (n = 11 – 13). Data are shown as the mean ± SEM. ns, not significant. (C) Blood glucose (n = 11 – 13). Data are shown as the mean ±

SEM. ns, not significant. (D) Bone mineral density (*n* = 11). Data are shown as the mean ± SEM. ns, not significant. (E) Grip strength (*n* = 11). Data are shown as the mean ± SEM. ns, not significant. (F) Hanging endurance on the RotaRod system before Que administration (*n* = 13). Data are shown as the mean ± SEM. ns, not significant. (G) Frequency of electric shock on the treadmill over 30 minutes before Que administration (*n* = 13). Data are shown as the mean ± SEM. ns, not significant. (H) Ejection fraction (EF), fractional shortening (FS), LV Vol;s, LV Vol;d and LV Mass AW (*n* = 12). Data are shown as the mean ± SEM. ns, not significant. (I) Lifespan (*n* = 13), ns, not significant. Y-Ctrl represents 10-week-old young male mice, and O-Veh and O-Que represent vehicle (10% PEG400 in PBS)- or low-dose Que-treated old male mice.

Figure S2. Organ weights and histological analysis in SKM, BAT and hearts were unaffected by Que. (A) Weights of 11 kinds of tissue (*n* = 5). Data are shown as the mean ± SEM. ns, not significant. (B) Histological analysis of SKM, BAT and hearts. Haematoxylin and eosin staining in SKM, BAT and hearts (left, *n* = 4). Scale bar, 100 µm. SA-β-Gal staining analysis in hearts (right, *n* = 4). Scale bar, 100 µm. Y-Ctrl represents 10-week-old young male mice, and O-Veh and O-Que represent vehicle (10% PEG400 in PBS)- or low-dose Que-treated old male mice.

Table S1. Primers used in this paper.

Mouse

Name	Sequence
L1-ORF1-F	ATCTGTCTCCCAGGTCTGCT
L1-ORF1-R	TCCTCCGTTTACCTTTCGCC
L1-3’UTR-F	AGCCAAATGGATGGACCTGG
L1-3’UTR-R	AAGGAGGGGCATAGTGTCCA
SINE B1-F	TGGCGCACGCCT TTA ATC
SINE B1-R	TGGCCTCGA ACTCAGAATCC
MLV5-F	TTCCCAATAAAGCCTCTTGC
MLV5-R	AGACCCTCCCAAGGATCAGC
LTR41-F	CCTCTCCACGGGTCTTGAAC
LTR41-R	TAGGGACCTCCGCTGATTGA
LTR42-F	CCACGGGTCTTGAACCTGAG
LTR42-R	CTCTAGGGACCTCCGCTGAT
IL6-F	ACCAGAGGAAATTTTCAATAGGC
IL6-R	TGATGCACTTGCAGAAAACA
β-actin-F	GGACTGTTACTGAGCTGCGTT
β-actin-R	CGCCTTCACCGTTCCAGTT

Human

Name	Sequence
L1-5’UTR-F	GCCAAGATGGCCGAATAGGA
L1-5’UTR-R	AAATCACCCGTCTTCTGCGT
L1-ORF1-F	ACCTGAAAGTGACGGGGAGA
L1-ORF1-R	CCTGCCTTGCTAGATTGGGG
Alu-F	CAACATAGTGAAACCCCGTCTCT

Alu-R	GCCTCAGCCTCCCGAGTAG
LTR10C-F	CAGCCAATGGAAACCGGACA
LTR10C-R	TTTTGCCACGAGAGTACACC
LTR2C-F	TGCTCGATCTATCACGACCC
LTR2C-R	ACTCCTCAGACACCGGAT
LTR35A-F	ACTACCTCACTGACGCCAT
LTR35A-R	TGGGACACTCCGCAAC
LTR3B-F	ACTGACAAACTGCGTCT
LTR3B-R	GCCCTTCGTATTTATTGGGT
IL6-F	ACTCACCTCTTCAGAACGAATTG
IL6-R	CCATCTTTGGAAGGTTCAGGTTG
β-actin-F	CATGTACGTTGCTATCCAGGC
β-actin-R	CTCCTTAATGTCACGCACGAT

Figure S1

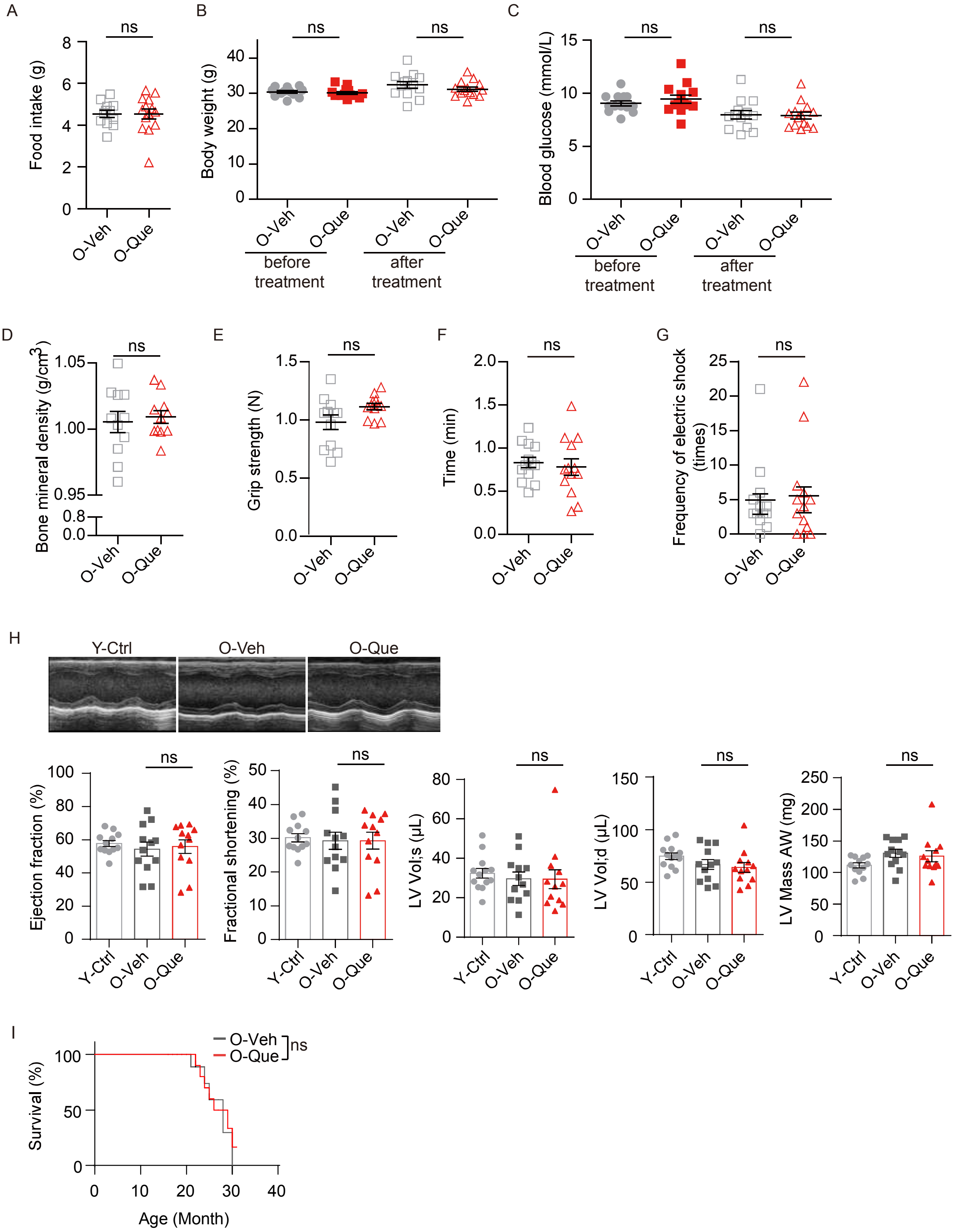
A
B
C
D
E
F
G
H
I
Food intake (g)
Body weight (g)
Blood glucose (mmol/L)
before treatment
after treatment
Bone mineral density (g/cm3)
Grip strength (N)
Time (min)
Frequency of electric shock (times)
Y-Ctrl
O-Veh
O-Que
Ejection fraction (%)
Fractional shortening (%)
LV Vol;s (μL)
LV Vol;d (μL)
LV Mass AW (mg)
Survival (%)
Age (Month)
ns

Figure S2

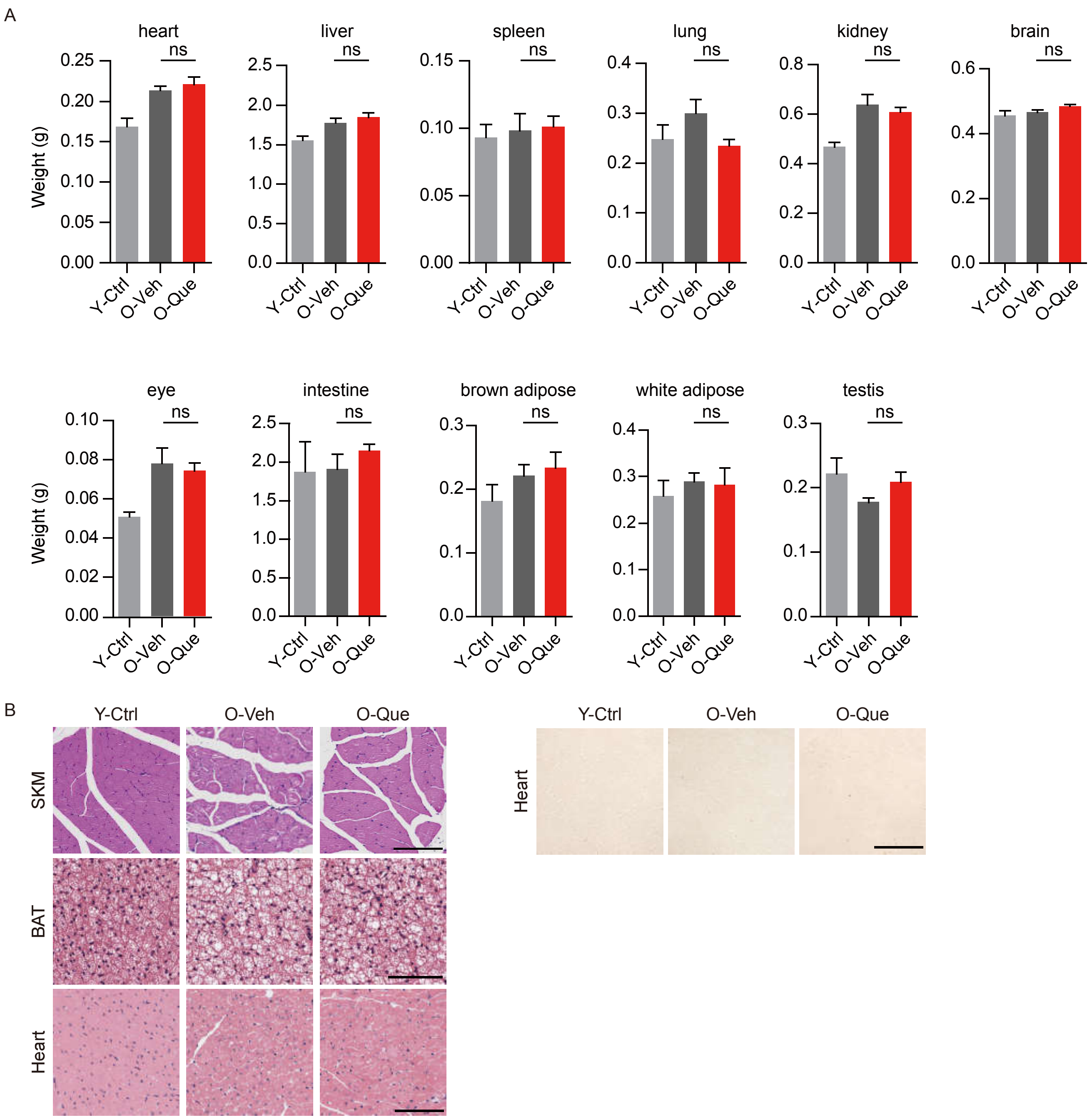
A
heart
liver
spleen
lung
kidney
brain
eye
intestine
brown adipose
white adipose
testis
ns
Weight (g)
Y-Ctrl
O-Veh
O-Que
B
SKM
BAT
Heart
